# 2,6-Diphenyl-4-(2-thien­yl)-1,4-dihydro­pyridine-3,5-dicarbonitrile

**DOI:** 10.1107/S1600536809033339

**Published:** 2009-08-26

**Authors:** Xiao-Tong Zhu, Ge Zhang, Ning Ma

**Affiliations:** a Department of Chemistry, Xuzhou Medical College, Jiangsu 221004, People’s Republic of China; b College of Chemistry and Chemical Engineering, Xuzhou Normal University, Xuzhou 221116, People’s Republic of China

## Abstract

The asymmetric unit of the title compound, C_23_H_15_N_3_S, contains two crystallographically independent mol­ecules. The pyridine rings adopt envelope conformations. The thio­phene rings are oriented at dihedral angles of 77.97 (4)/53.53 (4) and 78.44 (4)/57.11 (4)° with respect to the phenyl rings, while the dihedral angles between the phenyl rings are 48.51 (4) and 44.49 (4)°. In the crystal structure, inter­molecular N—H⋯N hydrogen bonds link the mol­ecules into chains along the *c* axis. The S, C and H atoms of one of the thio­phene rings are disordered over two orientations, with occupancy ratios of 0.314 (15):0.686 (15).

## Related literature

For general background to the synthesis of pyridines with a multi-aryl substitution pattern, see: Adib *et al.* (2006 ▶); Cave & Raston (2000 ▶); Kobayashi *et al.* (1991 ▶); Kröhnke (1963 ▶, 1976 ▶); Kumar *et al.* (2006 ▶); Tu *et al.* (2005*a* ▶,*b* ▶).
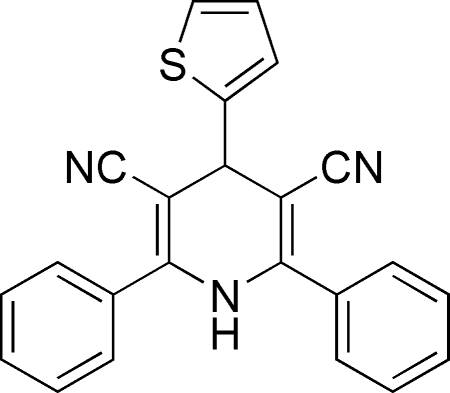


## Experimental

### 

#### Crystal data

C_23_H_15_N_3_S
*M* *_r_* = 365.44
Triclinic, 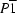

*a* = 11.2726 (14) Å
*b* = 11.8903 (15) Å
*c* = 14.5544 (17) Å
α = 86.571 (2)°
β = 88.755 (2)°
γ = 81.249 (1)°
*V* = 1924.5 (4) Å^3^
*Z* = 4
Mo *K*α radiation
μ = 0.18 mm^−1^
*T* = 298 K
0.40 × 0.31 × 0.30 mm

#### Data collection

Bruker SMART CCD area-detector diffractometer
Absorption correction: multi-scan (*SADABS*; Bruker, 2001 ▶) *T*_min_ = 0.932, *T*_max_ = 0.948
10076 measured reflections
6647 independent reflections
3520 reflections with *I* > 2σ(*I*)
*R*_int_ = 0.026

#### Refinement

*R*[*F* ^2^ > 2σ(*F* ^2^)] = 0.068
*wR*(*F* ^2^) = 0.199
*S* = 1.07
6647 reflections
524 parameters
H-atom parameters constrained
Δρ_max_ = 0.57 e Å^−3^
Δρ_min_ = −0.50 e Å^−3^

### 

Data collection: *SMART* (Bruker, 2001 ▶); cell refinement: *SAINT* (Bruker, 2001 ▶); data reduction: *SAINT*; program(s) used to solve structure: *SHELXS97* (Sheldrick, 2008 ▶); program(s) used to refine structure: *SHELXL97* (Sheldrick, 2008 ▶); molecular graphics: *SHELXTL* (Sheldrick, 2008 ▶); software used to prepare material for publication: *SHELXTL*.

## Supplementary Material

Crystal structure: contains datablocks global, I. DOI: 10.1107/S1600536809033339/hk2755sup1.cif

Structure factors: contains datablocks I. DOI: 10.1107/S1600536809033339/hk2755Isup2.hkl

Additional supplementary materials:  crystallographic information; 3D view; checkCIF report

## Figures and Tables

**Table 1:** Hydrogen-bond geometry (Å, °)

*D*—H⋯*A*	*D*—H	H⋯*A*	*D*⋯*A*	*D*—H⋯*A*
N1—H1⋯N5	0.86	2.10	2.956 (3)	173
N4—H4⋯N2^i^	0.86	2.19	3.042 (3)	172

Symmetry code: (i) 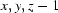.

**Fig. 1.**
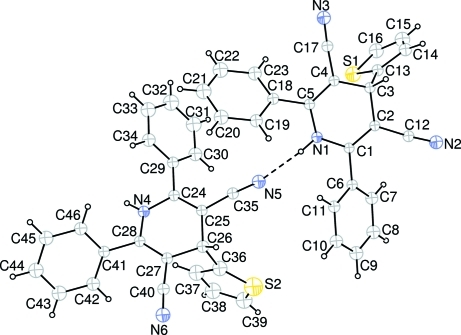
The molecular structure of the title molecule with the atom-numbering scheme. Hydrogen bond is shown as dashed line.

**Fig. 2.**
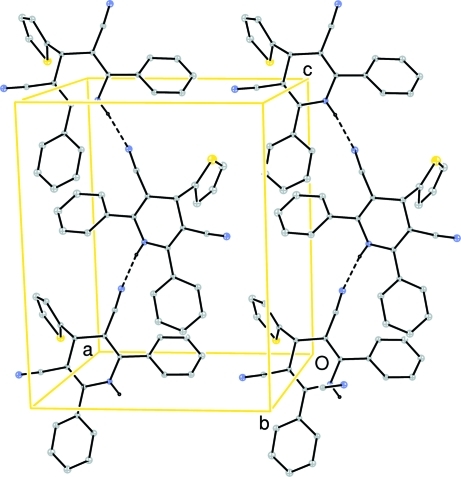
A partial packing diagram. Hydrogen bonds are shown as dashed lines. Hydrogen atoms not involved in hydrogen bonding are omitted for clarity.

## supplementary crystallographic information

### Comment

Pyridines are of interest because of the occurrence of their saturated and partially saturated derivatives in biologically active compounds and natural products such as NAD nucleotides, pyridoxol (vitamin B6), and pyridine alkaloids. Pyridines with a multi-aryl substitution pattern have been synthesized using various methods and procedures. Traditionally, these compounds have been synthesized through the reaction of *N*-phenacylpyridinium salts with unsaturated ketones in the presence of ammonium acetate (Kröhnke, 1963; Kröhnke, 1976). More recently, several new improved methods and procedures have been developed for the syntheses of these pyridines (Kobayashi *et al.*, 1991; Kumar *et al.* 2006), including solvent-free condition (Adib *et al.*, 2006; Cave & Raston, 2000) and microwave heating (Tu *et al.*, 2005a; Tu *et al.*, 2005b). We report herein the crystal structure of the title compound.

The asymmetric unit of the title compound contains two crystallographically independent molecules, in which they are linked through the intramolecular N-H···N hydrogen bond (Table 1, Fig. 1). The pyridine rings A (N1/C1-C5) and E (N4/C24-C28) adopt envelope conformations with atoms C3 and C26 displaced by 0.180 (3) and 0.238 (3) Å from the planes of the other ring atoms, respectively. Rings B (C6-C11), C (S1/C13-C16), D (C18-C23) and F (C29-C34), G (S2/C36-C39), H (C41-C46) are, of course, planar and they are oriented at dihedral angles of B/C = 77.97 (4), B/D = 48.51 (4), C/D = 53.53 (4) ° and F/G = 78.44 (4), F/H = 44.49 (4) °, G/H = 57.11 (4) °.

In the crystal structure, intra- and intermolecular N-H···N hydrogen bonds (Table 1) link the molecules into chains along the c axis (Fig. 2), in which they may be effective in the stabilization of the structure.

### Experimental

The title compound was prepared in 10-ml vial, 3-oxo-3-phenylpropanenitrile (0.15 g, 1 mmol), thiophene-2-carbaldehyde (0.11 g, 1 mmol), ammonium acetate (0.5 g) and water (2.0 ml) were mixed, and then capped. The mixture was irradiated for 16 min at 423 K (initial power 150 W and maximum power 250 W).

### Refinement

H atoms were positioned geometrically with N-H = 0.86 Å (for NH) and C-H = 0.93 and 0.98 Å for aromatic and methine H atoms, respectively, and constrained to ride on their parent atoms, with U_iso_(H) = 1.2U_eq_(C,N). S2, C37, C38, C39, H37, H38 and H39 atoms of the thiophene ring attached at C26 are disordered over two orientations. During the refinement process, the disordered S2, C37, C38, C39, H37, H38, H39 and S2', C37', C38', C39', H37', H38', H39' atoms were refined with occupancies of 0.314 (15) and 0.686 (15), respectively.

### Crystal data

**Table:**

C_23_H_15_N_3_S	*Z* = 4
*M**_r_* = 365.44	*F*(000) = 760
Triclinic, *P*1	*D*_x_ = 1.261 Mg m^−^^3^
Hall symbol: -P 1	Mo *K*α radiation, λ = 0.71073 Å
*a* = 11.2726 (14) Å	Cell parameters from 2139 reflections
*b* = 11.8903 (15) Å	θ = 2.3–24.9°
*c* = 14.5544 (17) Å	µ = 0.18 mm^−^^1^
α = 86.571 (2)°	*T* = 298 K
β = 88.755 (2)°	Block, yellow
γ = 81.249 (1)°	0.40 × 0.31 × 0.30 mm
*V* = 1924.5 (4) Å^3^	

### Data collection

**Table:**

Bruker SMART CCD area-detector diffractometer	6647 independent reflections
Radiation source: fine-focus sealed tube	3520 reflections with *I* > 2σ(*I*)
graphite	*R*_int_ = 0.026
φ and ω scans	θ_max_ = 25.0°, θ_min_ = 1.7°
Absorption correction: multi-scan (*SADABS*; Bruker, 2001)	*h* = −8→13
*T*_min_ = 0.932, *T*_max_ = 0.948	*k* = −12→14
10076 measured reflections	*l* = −15→17

### Refinement

**Table:**

Refinement on *F*^2^	Primary atom site location: structure-invariant direct methods
Least-squares matrix: full	Secondary atom site location: difference Fourier map
*R*[*F*^2^ > 2σ(*F*^2^)] = 0.068	Hydrogen site location: inferred from neighbouring sites
*wR*(*F*^2^) = 0.199	H-atom parameters constrained
*S* = 1.07	*w* = 1/[σ^2^(*F*_o_^2^) + (0.0913*P*)^2^] where *P* = (*F*_o_^2^ + 2*F*_c_^2^)/3
6647 reflections	(Δ/σ)_max_ = 0.001
524 parameters	Δρ_max_ = 0.57 e Å^−^^3^
0 restraints	Δρ_min_ = −0.50 e Å^−^^3^

### Special details

**Table:**

Geometry. All e.s.d.'s (except the e.s.d. in the dihedral angle between two l.s. planes) are estimated using the full covariance matrix. The cell e.s.d.'s are taken into account individually in the estimation of e.s.d.'s in distances, angles and torsion angles; correlations between e.s.d.'s in cell parameters are only used when they are defined by crystal symmetry. An approximate (isotropic) treatment of cell e.s.d.'s is used for estimating e.s.d.'s involving l.s. planes.
Refinement. Refinement of *F*^2^ against ALL reflections. The weighted *R*-factor *wR* and goodness of fit *S* are based on *F*^2^, conventional *R*-factors *R* are based on *F*, with *F* set to zero for negative *F*^2^. The threshold expression of *F*^2^ > σ(*F*^2^) is used only for calculating *R*-factors(gt) *etc*. and is not relevant to the choice of reflections for refinement. *R*-factors based on *F*^2^ are statistically about twice as large as those based on *F*, and *R*- factors based on ALL data will be even larger.

### Fractional atomic coordinates and isotropic or equivalent isotropic displacement parameters (Å^2^)

**Table:**

	*x*	*y*	*z*	*U*_iso_*/*U*_eq_	Occ. (<1)
S1	1.15432 (13)	0.07649 (11)	1.06543 (10)	0.0921 (5)	
S2	0.4086 (13)	0.1479 (9)	0.7278 (7)	0.133 (4)	0.314 (15)
S2'	0.4830 (7)	0.0834 (5)	0.5978 (5)	0.131 (2)	0.686 (15)
N1	0.8726 (3)	0.2896 (2)	0.94731 (17)	0.0443 (7)	
H1	0.8320	0.2821	0.8993	0.053*	
N2	0.8246 (3)	0.2069 (3)	1.2711 (2)	0.0662 (9)	
N3	1.2341 (3)	0.4398 (3)	0.9984 (2)	0.0646 (9)	
N4	0.6938 (3)	0.2732 (2)	0.44938 (17)	0.0507 (8)	
H4	0.7373	0.2546	0.4018	0.061*	
N5	0.7532 (3)	0.2538 (3)	0.7749 (2)	0.0673 (10)	
N6	0.2860 (3)	0.4465 (3)	0.4966 (2)	0.0709 (10)	
C1	0.8299 (3)	0.2556 (3)	1.0316 (2)	0.0402 (8)	
C2	0.8961 (3)	0.2574 (3)	1.1076 (2)	0.0407 (8)	
C3	1.0226 (3)	0.2887 (3)	1.1043 (2)	0.0428 (8)	
H3	1.0246	0.3478	1.1480	0.051*	
C4	1.0467 (3)	0.3400 (3)	1.0091 (2)	0.0418 (8)	
C5	0.9770 (3)	0.3352 (3)	0.9359 (2)	0.0429 (8)	
C6	0.7073 (3)	0.2246 (3)	1.0299 (2)	0.0441 (8)	
C7	0.6763 (4)	0.1307 (3)	1.0811 (2)	0.0599 (10)	
H7	0.7327	0.0854	1.1184	0.072*	
C8	0.5609 (4)	0.1051 (4)	1.0764 (3)	0.0722 (12)	
H8	0.5401	0.0421	1.1103	0.087*	
C9	0.4776 (4)	0.1718 (4)	1.0224 (3)	0.0711 (12)	
H9	0.4001	0.1542	1.0202	0.085*	
C10	0.5065 (4)	0.2637 (3)	0.9715 (3)	0.0623 (11)	
H10	0.4493	0.3087	0.9347	0.075*	
C11	0.6212 (3)	0.2898 (3)	0.9749 (2)	0.0514 (9)	
H11	0.6411	0.3523	0.9397	0.062*	
C12	0.8522 (3)	0.2293 (3)	1.1969 (2)	0.0472 (9)	
C13	1.1162 (3)	0.1895 (3)	1.1329 (2)	0.0514 (9)	
C14	1.1826 (4)	0.1745 (3)	1.2162 (3)	0.0629 (11)	
H14	1.1763	0.2254	1.2628	0.075*	
C15	1.2610 (4)	0.0671 (5)	1.2155 (4)	0.0915 (16)	
H15	1.3123	0.0400	1.2638	0.110*	
C16	1.2544 (4)	0.0088 (4)	1.1400 (4)	0.0870 (15)	
H16	1.3005	−0.0614	1.1304	0.104*	
C17	1.1503 (4)	0.3958 (3)	1.0003 (2)	0.0466 (9)	
C18	0.9985 (3)	0.3797 (3)	0.8404 (2)	0.0482 (9)	
C19	0.9072 (4)	0.4475 (3)	0.7920 (2)	0.0568 (10)	
H19	0.8337	0.4696	0.8210	0.068*	
C20	0.9240 (5)	0.4825 (3)	0.7014 (3)	0.0739 (13)	
H20	0.8623	0.5282	0.6694	0.089*	
C21	1.0329 (6)	0.4495 (4)	0.6583 (3)	0.0845 (15)	
H21	1.0442	0.4724	0.5969	0.101*	
C22	1.1238 (5)	0.3836 (4)	0.7049 (3)	0.0860 (15)	
H22	1.1972	0.3627	0.6755	0.103*	
C23	1.1080 (4)	0.3474 (4)	0.7958 (3)	0.0678 (11)	
H23	1.1703	0.3016	0.8271	0.081*	
C24	0.7442 (3)	0.2505 (3)	0.5357 (2)	0.0468 (9)	
C25	0.6731 (3)	0.2670 (3)	0.6107 (2)	0.0456 (9)	
C26	0.5381 (3)	0.2973 (3)	0.6072 (2)	0.0557 (10)	
H26	0.5144	0.3601	0.6477	0.067*	
C27	0.5019 (3)	0.3403 (3)	0.5100 (2)	0.0476 (9)	
C28	0.5775 (3)	0.3242 (3)	0.4368 (2)	0.0453 (8)	
C29	0.8750 (3)	0.2102 (3)	0.5369 (2)	0.0514 (9)	
C30	0.9234 (4)	0.1189 (4)	0.5951 (3)	0.0726 (12)	
H30	0.8735	0.0818	0.6341	0.087*	
C31	1.0459 (5)	0.0831 (5)	0.5952 (3)	0.0967 (16)	
H31	1.0781	0.0209	0.6333	0.116*	
C32	1.1197 (5)	0.1388 (6)	0.5395 (4)	0.1023 (17)	
H32	1.2023	0.1158	0.5410	0.123*	
C33	1.0726 (4)	0.2285 (5)	0.4814 (3)	0.0879 (15)	
H33	1.1232	0.2655	0.4430	0.106*	
C34	0.9508 (4)	0.2643 (4)	0.4795 (3)	0.0677 (11)	
H34	0.9193	0.3250	0.4396	0.081*	
C35	0.7223 (3)	0.2582 (3)	0.7005 (2)	0.0505 (9)	
C36	0.4766 (4)	0.2004 (5)	0.6415 (3)	0.0850 (15)	
C37	0.492 (4)	0.111 (4)	0.568 (3)	0.097 (11)	0.314 (15)
H37	0.5270	0.1204	0.5100	0.117*	0.314 (15)
C37'	0.4036 (13)	0.2143 (13)	0.7245 (9)	0.104 (4)	0.686 (15)
H37'	0.3899	0.2784	0.7590	0.125*	0.686 (15)
C38	0.442 (2)	0.008 (3)	0.6026 (17)	0.106 (9)	0.314 (15)
H38	0.4446	−0.0579	0.5711	0.128*	0.314 (15)
C38'	0.3546 (15)	0.1055 (15)	0.7434 (10)	0.123 (4)	0.686 (15)
H38'	0.3035	0.0924	0.7927	0.147*	0.686 (15)
C39	0.390 (16)	0.025 (15)	0.691 (11)	0.11 (3)	0.314 (15)
H39	0.3508	−0.0272	0.7244	0.130*	0.314 (15)
C39'	0.395 (7)	0.024 (7)	0.678 (5)	0.115 (18)	0.686 (15)
H39'	0.3765	−0.0495	0.6782	0.138*	0.686 (15)
C40	0.3836 (4)	0.3998 (3)	0.4991 (2)	0.0507 (9)	
C41	0.5466 (3)	0.3632 (3)	0.3408 (2)	0.0475 (9)	
C42	0.4461 (4)	0.3353 (3)	0.2997 (3)	0.0656 (11)	
H42	0.3964	0.2914	0.3328	0.079*	
C43	0.4195 (5)	0.3726 (4)	0.2094 (3)	0.0792 (14)	
H43	0.3510	0.3552	0.1823	0.095*	
C44	0.4940 (5)	0.4350 (4)	0.1603 (3)	0.0795 (15)	
H44	0.4766	0.4586	0.0993	0.095*	
C45	0.5935 (5)	0.4630 (3)	0.1995 (3)	0.0716 (13)	
H45	0.6432	0.5062	0.1657	0.086*	
C46	0.6204 (4)	0.4267 (3)	0.2904 (2)	0.0592 (10)	
H46	0.6884	0.4454	0.3172	0.071*	

### Atomic displacement parameters (Å^2^)

**Table:**

	*U*^11^	*U*^22^	*U*^33^	*U*^12^	*U*^13^	*U*^23^
S1	0.0863 (10)	0.0734 (8)	0.1132 (10)	0.0033 (7)	−0.0117 (8)	−0.0120 (7)
S2	0.149 (8)	0.130 (10)	0.123 (6)	−0.051 (8)	0.024 (5)	0.031 (7)
S2'	0.164 (4)	0.096 (4)	0.148 (5)	−0.078 (3)	−0.014 (4)	0.014 (3)
N1	0.0490 (18)	0.0587 (17)	0.0283 (14)	−0.0186 (15)	−0.0033 (12)	0.0004 (12)
N2	0.060 (2)	0.099 (3)	0.0362 (18)	−0.0057 (19)	0.0060 (15)	0.0059 (16)
N3	0.057 (2)	0.078 (2)	0.061 (2)	−0.017 (2)	0.0030 (17)	−0.0001 (17)
N4	0.052 (2)	0.0696 (19)	0.0288 (15)	−0.0018 (16)	−0.0001 (13)	−0.0033 (13)
N5	0.086 (3)	0.082 (2)	0.0387 (18)	−0.027 (2)	−0.0131 (17)	−0.0002 (15)
N6	0.059 (2)	0.089 (3)	0.061 (2)	−0.001 (2)	−0.0012 (18)	0.0019 (18)
C1	0.044 (2)	0.0445 (19)	0.0316 (18)	−0.0061 (16)	0.0029 (15)	−0.0032 (14)
C2	0.043 (2)	0.050 (2)	0.0287 (17)	−0.0051 (16)	0.0019 (15)	−0.0001 (14)
C3	0.048 (2)	0.049 (2)	0.0325 (17)	−0.0087 (17)	−0.0016 (15)	−0.0049 (14)
C4	0.044 (2)	0.0458 (19)	0.0361 (18)	−0.0089 (17)	0.0008 (16)	−0.0025 (14)
C5	0.048 (2)	0.0453 (19)	0.0368 (18)	−0.0106 (17)	0.0044 (16)	−0.0031 (14)
C6	0.046 (2)	0.055 (2)	0.0321 (17)	−0.0134 (18)	0.0011 (15)	0.0000 (15)
C7	0.060 (3)	0.074 (3)	0.047 (2)	−0.020 (2)	−0.0070 (19)	0.0119 (19)
C8	0.070 (3)	0.084 (3)	0.068 (3)	−0.037 (3)	−0.004 (2)	0.015 (2)
C9	0.057 (3)	0.095 (3)	0.067 (3)	−0.029 (3)	−0.002 (2)	−0.003 (2)
C10	0.051 (3)	0.073 (3)	0.063 (3)	−0.008 (2)	−0.008 (2)	−0.002 (2)
C11	0.050 (2)	0.058 (2)	0.046 (2)	−0.0087 (19)	0.0017 (17)	0.0020 (17)
C12	0.047 (2)	0.060 (2)	0.033 (2)	−0.0029 (18)	−0.0006 (16)	−0.0023 (16)
C13	0.047 (2)	0.055 (2)	0.053 (2)	−0.0143 (19)	−0.0028 (17)	0.0066 (17)
C14	0.056 (3)	0.072 (3)	0.056 (2)	0.001 (2)	−0.017 (2)	0.0124 (19)
C15	0.070 (3)	0.109 (4)	0.086 (4)	0.000 (3)	−0.018 (3)	0.041 (3)
C16	0.073 (3)	0.068 (3)	0.112 (4)	0.002 (3)	0.003 (3)	0.028 (3)
C17	0.049 (2)	0.052 (2)	0.0379 (19)	−0.007 (2)	0.0013 (17)	0.0018 (15)
C18	0.061 (3)	0.052 (2)	0.0356 (19)	−0.0237 (19)	0.0055 (18)	−0.0029 (15)
C19	0.074 (3)	0.059 (2)	0.039 (2)	−0.021 (2)	−0.0046 (19)	0.0040 (17)
C20	0.112 (4)	0.068 (3)	0.046 (2)	−0.030 (3)	−0.013 (3)	0.0098 (19)
C21	0.127 (5)	0.090 (3)	0.044 (2)	−0.046 (3)	0.007 (3)	0.007 (2)
C22	0.097 (4)	0.114 (4)	0.053 (3)	−0.035 (3)	0.030 (3)	−0.009 (3)
C23	0.071 (3)	0.086 (3)	0.046 (2)	−0.015 (2)	0.011 (2)	−0.002 (2)
C24	0.054 (2)	0.057 (2)	0.0306 (18)	−0.0107 (18)	−0.0064 (16)	0.0010 (15)
C25	0.057 (2)	0.052 (2)	0.0295 (18)	−0.0136 (18)	−0.0056 (16)	0.0013 (14)
C26	0.063 (3)	0.069 (2)	0.0342 (19)	−0.007 (2)	0.0037 (17)	−0.0001 (17)
C27	0.052 (2)	0.054 (2)	0.0367 (19)	−0.0095 (19)	−0.0072 (17)	0.0033 (15)
C28	0.049 (2)	0.052 (2)	0.0349 (19)	−0.0094 (18)	−0.0035 (16)	0.0006 (15)
C29	0.054 (2)	0.067 (2)	0.0339 (19)	−0.010 (2)	−0.0088 (17)	−0.0008 (16)
C30	0.064 (3)	0.089 (3)	0.059 (3)	0.002 (3)	−0.008 (2)	0.012 (2)
C31	0.078 (4)	0.121 (4)	0.080 (3)	0.016 (3)	−0.015 (3)	0.015 (3)
C32	0.061 (3)	0.153 (5)	0.088 (4)	0.005 (4)	−0.008 (3)	−0.012 (4)
C33	0.058 (3)	0.133 (4)	0.074 (3)	−0.020 (3)	−0.003 (2)	−0.001 (3)
C34	0.059 (3)	0.087 (3)	0.057 (2)	−0.012 (2)	−0.007 (2)	0.001 (2)
C35	0.061 (2)	0.059 (2)	0.035 (2)	−0.0192 (19)	−0.0027 (17)	0.0023 (16)
C36	0.066 (3)	0.114 (4)	0.071 (3)	−0.021 (3)	−0.011 (2)	0.051 (3)
C37	0.098 (17)	0.09 (2)	0.11 (2)	−0.041 (16)	0.004 (15)	0.040 (15)
C37'	0.117 (8)	0.092 (8)	0.092 (6)	−0.014 (8)	0.030 (5)	0.059 (7)
C38	0.108 (18)	0.11 (2)	0.103 (17)	−0.028 (15)	0.010 (14)	0.017 (15)
C38'	0.108 (10)	0.121 (11)	0.138 (11)	−0.038 (9)	0.004 (8)	0.050 (8)
C39	0.11 (5)	0.11 (7)	0.11 (4)	−0.04 (4)	−0.02 (3)	0.03 (4)
C39'	0.11 (3)	0.11 (3)	0.13 (3)	−0.05 (2)	−0.03 (2)	0.06 (2)
C40	0.056 (3)	0.060 (2)	0.0370 (19)	−0.012 (2)	0.0009 (18)	0.0019 (16)
C41	0.056 (2)	0.053 (2)	0.0319 (18)	−0.0024 (19)	−0.0012 (17)	−0.0003 (15)
C42	0.069 (3)	0.081 (3)	0.046 (2)	−0.007 (2)	−0.012 (2)	−0.0040 (19)
C43	0.084 (3)	0.098 (3)	0.051 (3)	0.009 (3)	−0.028 (2)	−0.008 (2)
C44	0.105 (4)	0.083 (3)	0.037 (2)	0.028 (3)	−0.010 (3)	0.003 (2)
C45	0.095 (4)	0.067 (3)	0.044 (2)	0.007 (3)	0.014 (2)	0.0105 (19)
C46	0.070 (3)	0.063 (2)	0.042 (2)	−0.004 (2)	0.0002 (19)	0.0006 (18)

### Geometric parameters (Å, °)

**Table:**

S1—C16	1.664 (5)	C21—C22	1.359 (7)
S1—C13	1.708 (4)	C21—H21	0.9300
S2—C36	1.603 (11)	C22—C23	1.382 (5)
S2—C39	1.62 (17)	C22—H22	0.9300
S2'—C36	1.555 (10)	C23—H23	0.9300
S2'—C39'	1.70 (8)	C24—C25	1.346 (5)
N1—C1	1.370 (4)	C24—C29	1.479 (5)
N1—C5	1.372 (4)	C25—C35	1.423 (5)
N1—H1	0.8600	C25—C26	1.511 (5)
N2—C12	1.145 (4)	C26—C36	1.488 (6)
N3—C17	1.146 (4)	C26—C27	1.518 (4)
N4—C28	1.369 (4)	C26—H26	0.9800
N4—C24	1.385 (4)	C27—C28	1.354 (5)
N4—H4	0.8600	C27—C40	1.419 (5)
N5—C35	1.140 (4)	C28—C41	1.477 (4)
N6—C40	1.155 (5)	C29—C34	1.384 (5)
C1—C2	1.350 (4)	C29—C30	1.387 (5)
C1—C6	1.484 (5)	C30—C31	1.381 (6)
C2—C12	1.421 (4)	C30—H30	0.9300
C2—C3	1.526 (5)	C31—C32	1.365 (7)
C3—C13	1.504 (5)	C31—H31	0.9300
C3—C4	1.516 (4)	C32—C33	1.370 (7)
C3—H3	0.9800	C32—H32	0.9300
C4—C5	1.346 (4)	C33—C34	1.374 (6)
C4—C17	1.429 (5)	C33—H33	0.9300
C5—C18	1.485 (4)	C34—H34	0.9300
C6—C11	1.383 (5)	C36—C37'	1.450 (15)
C6—C7	1.390 (5)	C36—C37	1.54 (6)
C7—C8	1.383 (5)	C37—C38	1.48 (5)
C7—H7	0.9300	C37—H37	0.9300
C8—C9	1.364 (5)	C37'—C38'	1.49 (3)
C8—H8	0.9300	C37'—H37'	0.9300
C9—C10	1.360 (5)	C38—C39	1.42 (15)
C9—H9	0.9300	C38—H38	0.9300
C10—C11	1.377 (5)	C38'—C39'	1.42 (7)
C10—H10	0.9300	C38'—H38'	0.9300
C11—H11	0.9300	C39—H39	0.9300
C13—C14	1.426 (5)	C39'—H39'	0.9300
C14—C15	1.439 (6)	C41—C46	1.377 (5)
C14—H14	0.9300	C41—C42	1.386 (5)
C15—C16	1.344 (7)	C42—C43	1.386 (5)
C15—H15	0.9300	C42—H42	0.9300
C16—H16	0.9300	C43—C44	1.366 (7)
C18—C19	1.384 (5)	C43—H43	0.9300
C18—C23	1.394 (5)	C44—C45	1.364 (7)
C19—C20	1.378 (5)	C44—H44	0.9300
C19—H19	0.9300	C45—C46	1.392 (5)
C20—C21	1.379 (7)	C45—H45	0.9300
C20—H20	0.9300	C46—H46	0.9300
			
C16—S1—C13	93.3 (3)	C24—C25—C35	121.1 (3)
C36—S2—C39	101 (6)	C24—C25—C26	123.9 (3)
C36—S2'—C39'	97 (3)	C35—C25—C26	115.0 (3)
C1—N1—C5	123.1 (3)	C36—C26—C25	112.0 (3)
C1—N1—H1	118.5	C36—C26—C27	112.5 (3)
C5—N1—H1	118.5	C25—C26—C27	109.1 (3)
C28—N4—C24	122.6 (3)	C36—C26—H26	107.7
C28—N4—H4	118.7	C25—C26—H26	107.7
C24—N4—H4	118.7	C27—C26—H26	107.7
C2—C1—N1	120.0 (3)	C28—C27—C40	120.9 (3)
C2—C1—C6	125.8 (3)	C28—C27—C26	122.6 (3)
N1—C1—C6	114.2 (3)	C40—C27—C26	116.5 (3)
C1—C2—C12	121.7 (3)	C27—C28—N4	120.1 (3)
C1—C2—C3	123.1 (3)	C27—C28—C41	124.8 (3)
C12—C2—C3	115.2 (3)	N4—C28—C41	115.0 (3)
C13—C3—C4	112.7 (3)	C34—C29—C30	119.1 (4)
C13—C3—C2	112.4 (3)	C34—C29—C24	119.8 (3)
C4—C3—C2	109.0 (3)	C30—C29—C24	121.0 (4)
C13—C3—H3	107.5	C31—C30—C29	120.0 (5)
C4—C3—H3	107.5	C31—C30—H30	120.0
C2—C3—H3	107.5	C29—C30—H30	120.0
C5—C4—C17	120.5 (3)	C32—C31—C30	120.2 (5)
C5—C4—C3	123.8 (3)	C32—C31—H31	119.9
C17—C4—C3	115.7 (3)	C30—C31—H31	119.9
C4—C5—N1	119.6 (3)	C31—C32—C33	120.2 (5)
C4—C5—C18	126.3 (3)	C31—C32—H32	119.9
N1—C5—C18	114.1 (3)	C33—C32—H32	119.9
C11—C6—C7	118.6 (3)	C32—C33—C34	120.4 (5)
C11—C6—C1	119.4 (3)	C32—C33—H33	119.8
C7—C6—C1	122.0 (3)	C34—C33—H33	119.8
C8—C7—C6	119.6 (4)	C33—C34—C29	120.1 (4)
C8—C7—H7	120.2	C33—C34—H34	119.9
C6—C7—H7	120.2	C29—C34—H34	119.9
C9—C8—C7	120.4 (4)	N5—C35—C25	174.9 (4)
C9—C8—H8	119.8	C37'—C36—C26	117.7 (8)
C7—C8—H8	119.8	C37'—C36—C37	133.3 (15)
C10—C9—C8	120.7 (4)	C26—C36—C37	108.1 (13)
C10—C9—H9	119.6	C37'—C36—S2'	115.5 (7)
C8—C9—H9	119.6	C26—C36—S2'	126.7 (5)
C9—C10—C11	119.5 (4)	C26—C36—S2	144.7 (6)
C9—C10—H10	120.2	C37—C36—S2	107.0 (12)
C11—C10—H10	120.2	S2'—C36—S2	87.6 (5)
C10—C11—C6	121.1 (3)	C38—C37—C36	110 (3)
C10—C11—H11	119.5	C38—C37—H37	125.0
C6—C11—H11	119.5	C36—C37—H37	125.0
N2—C12—C2	175.2 (4)	C36—C37'—C38'	106.5 (13)
C14—C13—C3	126.9 (3)	C36—C37'—H37'	126.8
C14—C13—S1	111.4 (3)	C38'—C37'—H37'	126.8
C3—C13—S1	121.8 (2)	C39—C38—C37	110 (8)
C13—C14—C15	108.1 (4)	C39—C38—H38	125.1
C13—C14—H14	126.0	C37—C38—H38	125.1
C15—C14—H14	126.0	C39'—C38'—C37'	112 (4)
C16—C15—C14	114.9 (4)	C39'—C38'—H38'	123.9
C16—C15—H15	122.5	C37'—C38'—H38'	123.9
C14—C15—H15	122.5	C38—C39—S2	112 (10)
C15—C16—S1	112.3 (4)	C38—C39—H39	124.0
C15—C16—H16	123.8	S2—C39—H39	124.0
S1—C16—H16	123.8	C38'—C39'—S2'	109 (5)
N3—C17—C4	176.3 (4)	C38'—C39'—H39'	125.6
C19—C18—C23	118.9 (3)	S2'—C39'—H39'	125.6
C19—C18—C5	120.4 (3)	N6—C40—C27	175.3 (4)
C23—C18—C5	120.6 (3)	C46—C41—C42	119.2 (3)
C20—C19—C18	120.6 (4)	C46—C41—C28	119.5 (3)
C20—C19—H19	119.7	C42—C41—C28	121.3 (3)
C18—C19—H19	119.7	C41—C42—C43	120.2 (4)
C19—C20—C21	119.6 (4)	C41—C42—H42	119.9
C19—C20—H20	120.2	C43—C42—H42	119.9
C21—C20—H20	120.2	C44—C43—C42	119.8 (4)
C22—C21—C20	120.5 (4)	C44—C43—H43	120.1
C22—C21—H21	119.8	C42—C43—H43	120.1
C20—C21—H21	119.8	C45—C44—C43	120.8 (4)
C21—C22—C23	120.5 (5)	C45—C44—H44	119.6
C21—C22—H22	119.7	C43—C44—H44	119.6
C23—C22—H22	119.7	C44—C45—C46	119.7 (4)
C22—C23—C18	119.8 (4)	C44—C45—H45	120.2
C22—C23—H23	120.1	C46—C45—H45	120.2
C18—C23—H23	120.1	C41—C46—C45	120.3 (4)
C25—C24—N4	118.9 (3)	C41—C46—H46	119.9
C25—C24—C29	125.3 (3)	C45—C46—H46	119.9
N4—C24—C29	115.8 (3)		
			
C5—N1—C1—C2	−5.5 (5)	C36—C26—C27—C28	−109.4 (4)
C5—N1—C1—C6	171.6 (3)	C25—C26—C27—C28	15.5 (5)
N1—C1—C2—C12	176.2 (3)	C36—C26—C27—C40	71.0 (4)
C6—C1—C2—C12	−0.5 (5)	C25—C26—C27—C40	−164.1 (3)
N1—C1—C2—C3	−4.1 (5)	C40—C27—C28—N4	175.7 (3)
C6—C1—C2—C3	179.2 (3)	C26—C27—C28—N4	−3.9 (5)
C1—C2—C3—C13	−113.5 (3)	C40—C27—C28—C41	−1.0 (5)
C12—C2—C3—C13	66.2 (4)	C26—C27—C28—C41	179.5 (3)
C1—C2—C3—C4	12.1 (4)	C24—N4—C28—C27	−9.1 (5)
C12—C2—C3—C4	−168.1 (3)	C24—N4—C28—C41	167.9 (3)
C13—C3—C4—C5	112.6 (4)	C25—C24—C29—C34	−134.3 (4)
C2—C3—C4—C5	−12.8 (4)	N4—C24—C29—C34	45.1 (5)
C13—C3—C4—C17	−67.3 (4)	C25—C24—C29—C30	45.4 (5)
C2—C3—C4—C17	167.2 (3)	N4—C24—C29—C30	−135.2 (4)
C17—C4—C5—N1	−174.7 (3)	C34—C29—C30—C31	−0.1 (6)
C3—C4—C5—N1	5.4 (5)	C24—C29—C30—C31	−179.8 (4)
C17—C4—C5—C18	2.5 (5)	C29—C30—C31—C32	1.4 (8)
C3—C4—C5—C18	−177.4 (3)	C30—C31—C32—C33	−1.8 (8)
C1—N1—C5—C4	4.9 (5)	C31—C32—C33—C34	0.9 (8)
C1—N1—C5—C18	−172.6 (3)	C32—C33—C34—C29	0.4 (7)
C2—C1—C6—C11	136.5 (4)	C30—C29—C34—C33	−0.8 (6)
N1—C1—C6—C11	−40.4 (4)	C24—C29—C34—C33	178.9 (4)
C2—C1—C6—C7	−44.3 (5)	C25—C26—C36—C37'	115.4 (7)
N1—C1—C6—C7	138.8 (3)	C27—C26—C36—C37'	−121.2 (7)
C11—C6—C7—C8	−0.3 (6)	C25—C26—C36—C37	−73.3 (19)
C1—C6—C7—C8	−179.5 (3)	C27—C26—C36—C37	50.0 (19)
C6—C7—C8—C9	−0.4 (6)	C25—C26—C36—S2'	−64.0 (6)
C7—C8—C9—C10	0.7 (7)	C27—C26—C36—S2'	59.3 (6)
C8—C9—C10—C11	−0.2 (6)	C25—C26—C36—S2	100.2 (11)
C9—C10—C11—C6	−0.6 (6)	C27—C26—C36—S2	−136.5 (10)
C7—C6—C11—C10	0.8 (5)	C39'—S2'—C36—C37'	−1(3)
C1—C6—C11—C10	180.0 (3)	C39'—S2'—C36—C26	178 (3)
C4—C3—C13—C14	127.8 (4)	C39'—S2'—C36—C37	−155 (7)
C2—C3—C13—C14	−108.6 (4)	C39'—S2'—C36—S2	7(3)
C4—C3—C13—S1	−53.0 (4)	C39—S2—C36—C37'	158 (7)
C2—C3—C13—S1	70.6 (3)	C39—S2—C36—C26	−174 (6)
C16—S1—C13—C14	−0.3 (3)	C39—S2—C36—C37	0(7)
C16—S1—C13—C3	−179.5 (3)	C39—S2—C36—S2'	−6(6)
C3—C13—C14—C15	179.2 (3)	C37'—C36—C37—C38	−16 (4)
S1—C13—C14—C15	0.0 (4)	C26—C36—C37—C38	175 (2)
C13—C14—C15—C16	0.4 (6)	S2'—C36—C37—C38	17 (3)
C14—C15—C16—S1	−0.6 (6)	S2—C36—C37—C38	−1(3)
C13—S1—C16—C15	0.5 (4)	C36—C37—C38—C39	3(8)
C4—C5—C18—C19	−131.2 (4)	C37—C38—C39—S2	−3(12)
N1—C5—C18—C19	46.1 (4)	C36—S2—C39—C38	2(11)
C4—C5—C18—C23	53.4 (5)	C26—C36—C37'—C38'	−179.3 (7)
N1—C5—C18—C23	−129.2 (4)	C37—C36—C37'—C38'	12 (3)
C23—C18—C19—C20	0.0 (5)	S2'—C36—C37'—C38'	0.2 (12)
C5—C18—C19—C20	−175.5 (3)	S2—C36—C37'—C38'	−17.5 (15)
C18—C19—C20—C21	0.2 (6)	C36—C37'—C38'—C39'	1(4)
C19—C20—C21—C22	−0.6 (7)	C37'—C38'—C39'—S2'	−2(5)
C20—C21—C22—C23	0.9 (7)	C36—S2'—C39'—C38'	2(5)
C21—C22—C23—C18	−0.7 (6)	C27—C28—C41—C46	128.1 (4)
C19—C18—C23—C22	0.3 (6)	N4—C28—C41—C46	−48.7 (4)
C5—C18—C23—C22	175.7 (3)	C27—C28—C41—C42	−53.3 (5)
C28—N4—C24—C25	7.7 (5)	N4—C28—C41—C42	129.9 (4)
C28—N4—C24—C29	−171.7 (3)	C46—C41—C42—C43	−1.0 (6)
N4—C24—C25—C35	−172.4 (3)	C28—C41—C42—C43	−179.7 (3)
C29—C24—C25—C35	7.0 (5)	C41—C42—C43—C44	1.4 (6)
N4—C24—C25—C26	6.8 (5)	C42—C43—C44—C45	−1.2 (7)
C29—C24—C25—C26	−173.8 (3)	C43—C44—C45—C46	0.7 (7)
C24—C25—C26—C36	108.1 (4)	C42—C41—C46—C45	0.5 (5)
C35—C25—C26—C36	−72.7 (4)	C28—C41—C46—C45	179.2 (3)
C24—C25—C26—C27	−17.1 (5)	C44—C45—C46—C41	−0.3 (6)
C35—C25—C26—C27	162.1 (3)		

### Hydrogen-bond geometry (Å, °)

**Table:**

*D*—H···*A*	*D*—H	H···*A*	*D*···*A*	*D*—H···*A*
N1—H1···N5	0.86	2.10	2.956 (3)	173
N4—H4···N2^i^	0.86	2.19	3.042 (3)	172

Symmetry codes: (i) *x*, *y*, *z*−1.
